# Heterogeneity among *Mycobacterium ulcerans* Isolates from Africa

**DOI:** 10.3201/eid1205.051191

**Published:** 2006-05

**Authors:** Pieter Stragier, Anthony Ablordey, L. Manou Bayonne, Yatta L. Lugor, Ireneaus S. Sindani, Patrick Suykerbuyk, Henry Wabinga, Wayne M. Meyers, Françoise Portaels

**Affiliations:** * Institute of Tropical Medicine, Antwerp, Belgium;; † Centre Hospitalier de Libreville, Libreville, Gabon;; ‡ Yambio Hospital, Eldoret, Kenya;; § World Health Organization South Sudan Office, Gigiri, Nairobi, Kenya;; ¶ Makerere University, Kampala, Uganda;; # Armed Forces Institute of Pathology, Washington, DC, USA

**Keywords:** Mycobacterium ulcerans, Buruli ulcer, genotyping, Africa, heterogeneity, dispatch

## Abstract

*Mycobacterium ulcerans* causes Buruli ulcer, an ulcerative skin disease in tropical and subtropical areas. Despite restricted genetic diversity, mycobacterial interspersed repetitive unit–variable-number tandem repeat analysis on *M. ulcerans* revealed 3 genotypes from different African countries. It is the first time this typing method succeeded directly on patient samples.

Buruli ulcer (BU), the third most common mycobacterial disease after tuberculosis and leprosy, is a major health problem in several West and Central African countries (*1*). Although endemic in Central America and subtropical climates of Southeast Asia and Australia, countries in Africa in the past decade have recorded increased incidence rates in some communities exceeding that of tuberculosis (*2*).

Mode(s) of transmission, natural reservoir(s) and other key aspects of the epidemiology of BU are not fully understood, a situation partly complicated by an apparent lack of genetic diversity of *Mycobacterium ulcerans*, as shown by several independent genetic markers (*3**–**6*). Conventional and molecular data suggest that *M. ulcerans* is an environmental pathogen because of the selective association of BU-endemic foci with wetlands and overflowed river banks and the detection of *M. ulcerans*–specific sequences in water, mud, aquatic insects, and plants (*7**–**9*). Specific reservoirs of the etiologic agent cannot be definitively assigned; however, we have cultivated *M. ulcerans* from a single aquatic insect from Benin (*10*).

Extensive molecular typing of *M. ulcerans* isolates recovered from patients in many endemic foci has been undertaken to further the understanding of the epidemiology of BU. A set of robust genotyping methods has already been applied to *M. ulcerans*: IS*2404* restriction fragment length polymorphism (*11*), amplified fragment length polymorphism analysis (AFLP) (*12*), multilocus sequence typing (*13*), variable-number tandem repeat (VNTR) (*3*), mycobacterial interspersed repetitive unit (MIRU)–VNTR (*6*), IS*2426* polymerase chain reaction (PCR) (*5*), and IS*2404*-Mtb2 PCR (*4*). All methods, except AFLP, resulted in geographically related genotypes for China, Japan, Mexico, Suriname, French Guiana, Malaysia, Papua New Guinea II and Papua New Guinea III, Australia Victoria, Australia Queensland, and Africa. Current typing methods have established a striking geographic and temporal homogeneity in African isolates from Angola, Benin, Democratic Republic of Congo (DRC), Ghana, Côte d'Ivoire, and Togo (*3**–**6*). Even *M. ulcerans* cultured from the insect collected in Benin showed an identical African genotype (*6*). Recently, however, Hilty et al., using a VNTR typing method and sequence analysis, described 3 genotypes in Ghana (*14*). The development of more discriminating typing methods may unravel the source and mode of transmission of *M. ulcerans* and other epidemiologic aspects of BU.

Improved understanding of the molecular biology of *M. ulcerans* will likely help elucidate observed differences in clinical manifestations. Reported disease recurrence rates vary from 6% to >20% (*15*). To what degree this recurrence is attributable to exogenous reinfection or dissemination of the pathogen from previous lesions is unknown. The relative contribution of variations in pathogen and host factors to progression and severity of disease likewise remains obscure.

We report the first evidence of genetic diversity in *M. ulcerans* samples from 3 African countries: DRC, Sudan, and Uganda. Previously, we identified tandem repeat loci, MIRUs (*6*), and VNTRs (*3*) in the genome of *M. ulcerans*. A selection of these MIRUs and VNTRs were used in this study to analyze *M. ulcerans* extracts from tissue specimens from Benin, Togo, Gabon, Uganda, and Sudan, and from previous isolates from patients from Cameroon, DRC, Uganda, and Congo-Brazzaville (Table 1). Results were compared with those of a geographically diverse collection (n = 39) that were typed in our previous study (*6*).

**Table 1:** MIRU-VNTR profiles of *Mycobacterium ulcerans* and origin of specimens (BK no.) or culture isolates*

ITM no./loci†	1‡	6‡	9‡	33‡	Genotype	Origin	Ziehl-Neelsen staining§	Year¶
5142	1	1	1	2	Victoria	Victoria, Australia		1967
9540	1	1	1	3	Southeast Asia	Queensland, Australia; PNG; Malaysia		1978
98-0912, 8756	1	2	1	3	Asia	China, Japan		1998
BK03-0621	2	1	1	3	PNGII	PNG	3+	2003
BK02-2487	2	1	1	1	PNGIII	PNG	1+	2002
BK04-0296	2	1	1	1		PNG	1+	2004
842	NA	1	2	1	Suriname	Suriname		1984
7922	2	2	2	1	French Guiana	French Guiana		1990
5114	1	2	2	1	Mexico	Mexico		1953
5116	1	2	2	2	Central African Congo River Basin	Maniema, DRC		1962
9099	1	2	2	2		Maniema, DRC		1964
5150	3	1	1	3	Atlantic Africa	Bas-Congo, DRC		1962
94-0662	3	1	1	3		Côte d'Ivoire		1994
96-0658	3	1	1	3		Angola		1996
97-0483	3	1	1	3		Ghana		1997
BK04-0875	3	1	1	3		Togo	4+	2004
BK04-1396	3	1	1	3		Benin	–	2004
02-0280	3	1	1	3		Cameroon		2002
02-1081	3	1	1	3		Cameroon		2002
05-0303	3	1	1	3		Congo-Brazzaville		1979
05-0304	3	1	1	3		Congo-Brazzaville		1979
BK05-0027	3	1	1	3		Gabon	1+	2005
BK04-1591	4	1	1	1	East African Nile River Basin	Sudan	4+	2004
BK04-1601	4	1	1	1		Sudan	–	2004
05-0861	4	1	1	1		Orientale, DRC		1959
05-1459	4	1	1	1		Uganda (NCTC no. 10445)		1964
BK04-0513	4	1	1	1		Uganda	1+	2004
BK05-0614	4	1	1	1		Uganda	4+	2005

*MIRU, mycobacterial interspersed repetitive unit; VNTR, variable-number tandem repeat; PNG, Papua New Guinea; DRC, Democratic Republic of Congo; NA, no amplification; NCTC, National Collection of Type Cultures. Shaded fields represent results from our previous study (*6*). †ITM numbers (Institute of Tropical Medicine). These numbers are representative members for the genotype each belongs to (*6*). ‡Numbers in columns 2 through 5 represent the number of repeats at the specific locus. These numbers form a pattern that divides *M. ulcerans* into genotypes. §Scale of the American Thoracic Society. Ziehl-Neelsen staining has not been done on culture isolates, since identifying acid-fast bacilli in a culture is an obsolete practice. ¶The date represents the year of isolation.

## The Study

To investigate the MIRU polymorphism, whole genomic DNA was prepared from bacterial cultures or clinical specimens. The specimens were tissue fragments from patients with nonulcerated (plaques and edematous forms) or ulcerated forms. DNA extraction from pure cultures was performed by heating the colonies in Tris-EDTA at 95°C for 10 minutes. Clinical specimens from laboratory-confirmed cases of BU were decontaminated by using the reversed Petroff method, and mycobacterial DNA was extracted from the decontaminated solution as previously described (*6*). Smears of the suspensions were stained by the Ziehl-Neelsen method.

PCR was run as previously described (*6*). The Agilent 2100 Bioanalyzer system (Agilent Technologies, Waldbronn, Germany) was used to separate 1 μL of PCR product electrophoretically.

Comparison of MIRU-VNTR copy numbers using 4 loci showed 11 different profiles. *M. ulcerans* isolates from DRC and Uganda and tissue extracts from patients from Sudan (Nzara) and Uganda (Nakasongola) showed distinct profiles (Central Africa: 1222 and East Africa: 4111), different from the originally homogeneous African genotype (Atlantic Africa: 3113; Table 1). In DRC, 3 different genotypes exist, corresponding to 3 different provinces: Bas-Congo, Maniema (Kasongo), and Orientale (Bunia). The isolate from Orientale was from near the Ugandan border (Lake Albert). Isolates from Gabon, Congo-Brazzaville, and Cameroon had the typical African genotype, now designated the Atlantic African genotype. Identical MIRU-VNTR profiles were observed by using DNA extracted from tissues or cultures from patients residing in the same area. The specificity of the MIRU-VNTR method was tested on 14 different *Mycobacterium* spp. Only *M. marinum*, *M. shottsii*, and *M. liflandii* tested positive, but they were distinguished from *M. ulcerans* by exhibiting different profiles (data not shown). Sequencing of the concerned loci showed the conserved MIRU sequence at locus 1 and 9 in *M. ulcerans*. Locus 6 (*3*) and locus 33 contain respectively a 56-bp and a 58-bp tandem repeat (Table 2).

**Table 2:** Primer sequence and location in *Mycobacterium ulcerans* and amplicon length at loci 1, 6, 9, and 33, resulting from a polymorphism in tandem repeat copy numbers

Locus	Primer sequence			Amplicon length			
	Forward primer (5´–3´)	Reverse primer (5´–3´)	Location	1 copy	2 copies	3 copies	4 copies
1	GCTGGTTCATGCGTGGAAG	GCCCTCGGGAATGTGGTT	mu0115C04F	380	433	486	539
6	GACCGTCATGTCGTTCGATCCTAGT	GACATCGAAGAGGTGTGCCGTCT	mu0019B07G	500	556	–	–
9	GCCGAAGCCTTGTTGGACG	GGTTTCCCGCAGCATCTCG	mu0113D07F	435	488	–	–
33	CAAGACTCCCACCGACAGGC	CGGATCGGCACGGTTCA	mu0043E11R	720	778	836	–

## Conclusions

Although *M. ulcerans* isolates from Africa are relatively homogeneous, this study demonstrates more heterogeneity between strains than previously reported. All isolates from West Africa (Côte d'Ivoire, Ghana, Togo, Benin) and Central Africa (Cameroon; Gabon; Congo-Brazzaville; DRC Bas-Congo; Angola) have the identical MIRU-VNTR profile, and all originate from regions (i.e., Bas-Congo) or countries that border the Atlantic Ocean. The isolates that come from regions or countries in the Nile River basin (i.e., Orientale in DRC, Sudan, and Uganda) or the Congo River basin (i.e., Maniema) have distinct profiles.

These results demonstrate for the first time heterogeneity among *M. ulcerans* from different African countries. The 3 African profiles are the Atlantic African profile, the Central African Congo River basin profile, and the East African Nile River basin profile. This is also the first detection of MIRUs and VNTRs in clinical specimens, even in smear-negative specimens.

These data show that MIRUs and VNTRs are helpful tools in genotyping *M. ulcerans*. Further detailed differentiation of this etiologic agent will lead to an understanding of the epidemiology of BU. As in tuberculosis, better discriminatory typing methods help assess the efficacy of antimycobacterial treatment of BU patients by differentiating reactivation from reinfection. Although *M. ulcerans* appears to be quite monomorphic, full sequencing of this organism will permit detection of genes specific for *M. ulcerans*, and more discriminatory VNTR should become available.
